# Profiling of conserved non-coding elements upstream of *SHOX* and functional characterisation of the *SHOX cis*-regulatory landscape

**DOI:** 10.1038/srep17667

**Published:** 2015-12-03

**Authors:** Hannah Verdin, Ana Fernández-Miñán, Sara Benito-Sanz, Sandra Janssens, Bert Callewaert, Kathleen De Waele, Jean De Schepper, Inge François, Björn Menten, Karen E. Heath, José Luis Gómez-Skarmeta, Elfride De Baere

**Affiliations:** 1 Center for Medical Genetics Ghent, Ghent University, Ghent, Belgium; 2 Centro Andaluz de Biología del Desarrollo, Consejo Superior de Investigaciones Científicas and Universidad Pablo de Olavide, Sevilla, Spain; 3 Institute of Medical and Molecular Genetics (INGEMM), Hospital Universitario La Paz, Universidad Autónoma de Madrid, IdiPAZ, Madrid, Spain; 4 Centro de Investigación Biomédica en Enfermedades Raras (CIBERER), Instituto Carlos III, Madrid, Spain; 5 Department of Pediatrics, Ghent University Hospital, Ghent, Belgium; 6 Department of Pediatric Endocrinology, University Hospitals Leuven, Leuven, Belgium

## Abstract

Genetic defects such as copy number variations (CNVs) in non-coding regions containing conserved non-coding elements (CNEs) outside the transcription unit of their target gene, can underlie genetic disease. An example of this is the short stature homeobox (*SHOX*) gene, regulated by seven CNEs located downstream and upstream of *SHOX*, with proven enhancer capacity in chicken limbs. CNVs of the downstream CNEs have been reported in many idiopathic short stature (ISS) cases, however, only recently have a few CNVs of the upstream enhancers been identified. Here, we set out to provide insight into: (i) the *cis*-regulatory role of these upstream CNEs in human cells, (ii) the prevalence of upstream CNVs in ISS, and (iii) the chromatin architecture of the *SHOX cis*-regulatory landscape in chicken and human cells. Firstly, luciferase assays in human U2OS cells, and 4C-seq both in chicken limb buds and human U2OS cells, demonstrated *cis*-regulatory enhancer capacities of the upstream CNEs. Secondly, CNVs of these upstream CNEs were found in three of 501 ISS patients. Finally, our 4C-seq interaction map of the *SHOX* region reveals a *cis*-regulatory domain spanning more than 1 Mb and harbouring putative new *cis*-regulatory elements.

The short stature homeobox-containing gene (*SHOX;* MIM *312865) is located in the pseudoautosomal region (PAR1) on the short arms of the X- and Y-chromosomes^1^. SHOX is a member of the paired-like homeobox-containing protein family of transcription factors, and plays a crucial role in bone development and growth^1^,^2^,^3^. A strict regulation of its spatiotemporal expression is therefore of utmost importance. As for most developmental genes, the minimal promoter region of *SHOX* is not sufficient but requires the assistance of *cis-*regulatory elements such as enhancers^4^. To date, seven evolutionarily conserved non-coding elements (abbreviated as CNEs, ECRs or ECS) located downstream (CNE4, CNE5, ECR1 and ECS4/CNE9) and upstream (CNE-5, CNE-3 and CNE-2) of *SHOX*, have been shown to act as enhancers^5^,^6^,^7^,^8^. As *SHOX* does not have an orthologue in rodents but is highly conserved in chicken^3^, and as *Shox* expression was shown in chicken limb buds at developmental stage HH26^6^, limb buds of chicken embryos have been used to confirm the enhancer potential of all CNEs, except for ECR1^6^,^7^. In addition, chromosome conformation capture (3C) in chicken embryo limbs demonstrated that the downstream ECR1 and CNE9 interact with the *Shox* promoter^8^. Interestingly, the regulatory capacity of CNE4, CNE5 and CNE9 was also investigated in whole zebrafish embryos resulting in the identification of additional tissues under the regulatory control of these CNEs and the identification of smaller, more deeply conserved sub-sequences within these CNEs^9^. Cotney *et al*. recently performed H3K27ac ChIP-seq profiling in human embryonic limb buds from day 33 (E33) through to E47^10^. This revealed a genome-wide distribution of the active enhancer-associated histone marks H3K27ac in human limbs of different developmental stages. Despite all this data, functional studies specifically investigating the *cis*-regulatory activity of the upstream CNEs in relevant human systems are lacking as only the enhancer potential of the four downstream CNEs was substantiated in human U2OS osteosarcoma cells.

Heterozygous defects of *SHOX* or its enhancers explain ~60–80% of cases with Leri-Weill dyschondrosteosis (LWD, MIM 127300) and ~2–5% of cases with idiopathic short stature (ISS, MIM 300582), which is defined as a height below −2 standard deviations (SDS) in the absence of known causes^11^. Molecular defects leading to both phenotypes include intragenic *SHOX* mutations, complete or partial *SHOX* deletions and duplications, and deletions and duplications of the downstream enhancer region^1^,^12^,^13^,^14^,^15^,^16^,^17^,^18^,^19^,^20^,^21^. Interestingly, only one deletion and duplication of the upstream enhancer region, have been described in ISS patients^21^,^22^.

Here, our aim was to provide insight into: (i) the *cis-*regulatory role of these upstream CNEs in relevant human cells, (ii) the prevalence of copy number variations (CNVs) of upstream CNEs in ISS, and (iii) the chromatin architecture of the *SHOX cis*-regulatory landscape in chicken and human cells. Luciferase assays of the upstream CNEs revealed enhancer activity in human U2OS cells. In addition, these CNEs overlap with H3K27ac ChIP-seq marks previously generated in human embryonic limb buds. Furthermore, 4C-seq in chicken embryo limb buds and in U2OS cells uncovered interactions of the upstream CNEs with the *Shox* promoter. CNVs of these upstream CNEs were found in only three of 501 patients with unexplained ISS.

## Results

### Luciferase assays of upstream CNEs in human U2OS osteosarcoma cells

**Figure 1:**
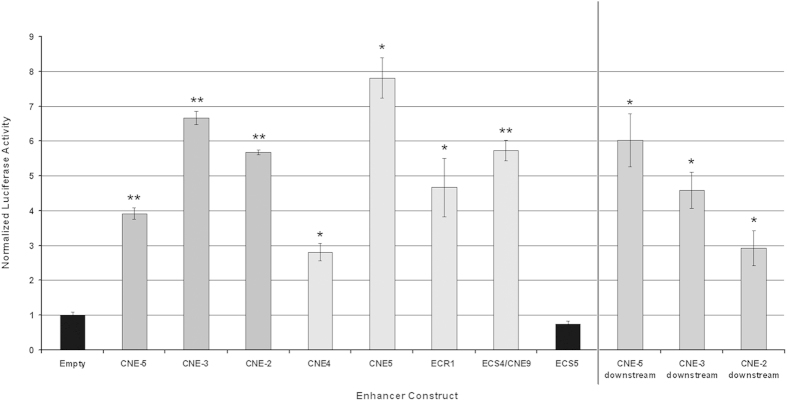
Enhancer activity of three upstream CNEs demonstrated by *in vitro* luciferase assays in human U2OS cells. Luciferase reporter activity of U2OS cells transfected with the pGL3SHOXprom enhancer reporter plasmids and the Renilla luciferase control plasmid. The pGL3SHOXprom without any enhancer inserted was used as an empty vector, to normalize luciferase activity. Fold increase values were obtained by normalizing the relative luciferase units of each sample, first, with respect to the Renilla luciferase activity, and second, to that transfected with the empty reporter plasmid. All values represent the mean and standard deviation of two biological replicates with each sample assayed in triplicate. ECS5, localized outside of the deleted region and which has previously been shown not to have enhancer activity, was utilized as a negative control^5^. The downstream *SHOX* enhancer regions CNE4, CNE5, ECR1 and CNE9/ECS4 were utilized as positive controls^6^,^8^. Increased luciferase activity was observed for all three upstream *SHOX* enhancer constructs, CNE-5, CNE-3 and CNE-2 constructs and the positive controls.

*In ovo* enhancer assays in chicken limb buds previously demonstrated that the upstream CNEs possess enhancer activity^7^. However, enhancer assays in relevant human cells have not yet been performed. Hence, luciferase assays of the upstream enhancers, CNE-5, CNE-3 and CNE-2, were carried out in human U2OS cells. For this purpose, the CNEs were cloned upstream of the *SHOX* promoter in the pGL3SHOXprom vector^8^, mimicking their chromosomal localization. The clones were co-transfected with a Renilla luciferase control vector into U2OS cells. The four downstream *SHOX* enhancer regions CNE4, CNE5, ECR1 and ECS4/CNE9 were used as positive controls whilst the pGL3SHOXprom vector with no enhancer sequence was used as the negative control. Increased luciferase activity was observed for all three upstream CNEs and positive controls (Fig. 1). The three upstream CNEs were also cloned downstream of the luciferase gene, to test out their localization dependency. All three showed enhancer activity (Fig. 1), demonstrating that the upstream CNEs act as enhancers in a localization independent manner in human U20S cells.

**Figure 2:**
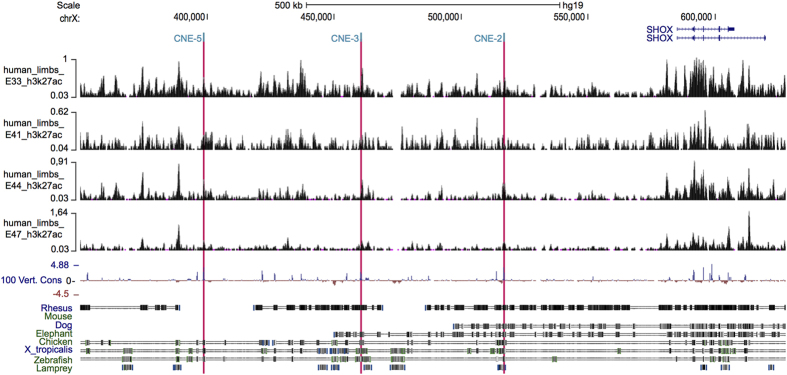
H3K27ac distribution of the *SHOX* locus in human embryonic limbs from embryonic day 33 (E33) through E47. Overview of the human *SHOX* locus (chrX:350,000–628,000; UCSC, Human Genome Browser, hg19), showing the distribution of enhancer-associated H3K27ac histone marks at different human embryonic limb bud stages, produced by Cotney *et al*. (2013) and retrieved from GEO dataset GSE42413. Four embryonic stages are represented: E33, E41, E44 and E47. Upstream enhancers CNE-5, CNE-3 and CNE-2 are highlighted with red bars. The two *SHOX* isoforms are represented in blue at the top. A conservation track is represented at the bottom.

Exploration of the *SHOX* region in H3K27ac ChIP-seq marks in human embryonic limb buds^10^ showed the presence of active marks in all three upstream CNEs (CNE-5, CNE-3 and CNE-2), substantiating their enhancer activity in the human developing limb (Fig. 2).

### Copy number profiling of upstream CNEs in cohort with unexplained ISS

#### CNV analysis of upstream CNEs

The study cohort consisted of 501 ISS patients. Deletions and duplications of *SHOX* and the downstream enhancer region were excluded prior to this study^18^, and intragenic mutations of *SHOX* were excluded in 56 of the 501 patients by Sanger sequencing of the coding regions. In the remaining 445 patients, coding mutations were excluded by a customized targeted amplicon-based next-generation sequencing approach^23^.

**Figure 3:**
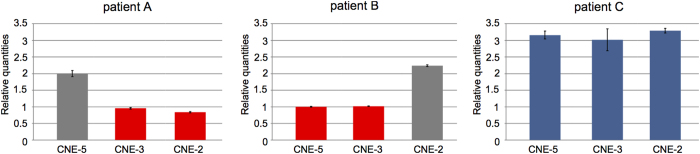
Copy number profiling of upstream CNEs (CNE-5, CNE-3 and CNE-2) revealing two upstream deletions and one duplication. Each panel shows the results of the CNV analysis using qPCR. The qPCR-derived copy number results are presented as relative quantities in a bar chart. Normal copy numbers are represented in grey, deletions are shown in red and duplications in blue. Error bars are added to allow interpretation of the assay’s precision.

**Figure 4:**
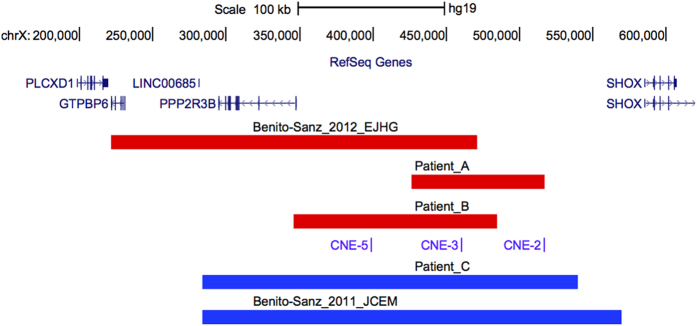
Overview of all reported upstream CNVs in the *SHOX* region. Overview of the *SHOX* region (hg19: chrX:151,000-628,000; UCSC, Human Genome Browser, hg19) with custom tracks showing the deletions (red bars) and duplications (blue bars) identified in this and previous studies^21^,^22^. A custom track representing the upstream enhancers (CNE-5, CNE-3 and CNE-2) is included, the RefSeq Genes track is shown at the top.

Overall, 501 patients with unexplained ISS underwent CNV analysis of the upstream enhancer region (CNE-5, CNE-3, CNE-2). A CNV of at least two of the upstream enhancers (two deletions, one duplication) was detected in three unrelated index cases (patients A, B and C) (Fig. 3). These CNVs were confirmed and delineated using customized high-resolution arrayCGH (Supplementary Fig. S1). An overview of these novel and previously reported upstream CNVs^21^,^22^ is given in Fig. 4. A summary of the sex, age, anthropometric and clinical details of the probands with these upstream CNVs and their family members is shown in Table 1.

In patient A, we found a deletion of ~91 kb encompassing CNE-3 and CNE-2 (Fig. 3). Patient A is a 9-year-old Belgian girl referred for ISS (115.8 cm, −2.3 height standard deviation score, SDS), with a reduced arm span:height ratio which is indicative of shortening of upper limbs and suggestive of LWD. Her parents’ heights were both within normal limits. The deletion was shown to be paternal (Supplementary Fig. S2).

In patient B, a deletion of ~139 kb was observed containing CNE-5 and CNE-3 (Fig. 3). Patient B is a 13-year-old boy of Roma origin referred for ISS (130.5 cm, −2.5 SDS). His father’s height is 170 cm (1.03 SDS) and his mother’s height is 152 cm (−1.96 SDS). Paternal inheritance of the deletion was excluded but no maternal material was available to substantiate a maternal origin (Supplementary Fig. S2).

A duplication of ~256 kb containing all three upstream CNEs was identified in patient C (Fig. 3). Patient C is a 17-year-old Belgian girl with disproportionate short stature (153 cm, −2.3 SDS). Her father´s height is 163 cm (−2.6 SDS) and her mother’s height is 156 cm (−1.8 SDS). The duplication was shown to be paternal (Supplementary Fig. S2).

#### Structural variations of the SHOX region in open source and local databases

No upstream CNVs were identified in a control population of 340 healthy individuals with heights within the normal range^21^,^22^. To further evaluate the prevalence and associated phenotypic effects of the upstream CNVs, we searched for deletions and duplications of these regions in the Database of Genomic Variants (DGV)^24^, Database of Chromosomal Imbalance and Phenotype in Humans Using Ensembl Resources (Decipher)^25^, and our local arrayCGH database. Four duplications of the upstream region were present in DGV^26^,^27^,^28^ while 19 duplications and one deletion were reported in Decipher. However, short stature was only listed as part of the phenotypic spectrum for one of the duplications in Decipher. This duplication was predicted to be benign as the patient had three other CNVs, one of which was classified as probably pathogenic. Similarly, the majority of the Decipher listed CNVs encompassing the upstream enhancers are classified as likely benign and occur in combination with other CNVs^25^ (Supplementary Table S3).

In our local array CGH database, containing genomic profiles from over 3000 patients, one deletion and two duplications of the upstream enhancers were found in patients with syndromic phenotypes. As two of these patients display short stature in combination with a variety of symptoms, the short stature may be part of a syndrome and caused by a different genetic defect (Supplementary Table S3).

#### Chromatin interaction map (4C-seq) in chicken limb buds and in human U2OS cells

**Figure 5:**
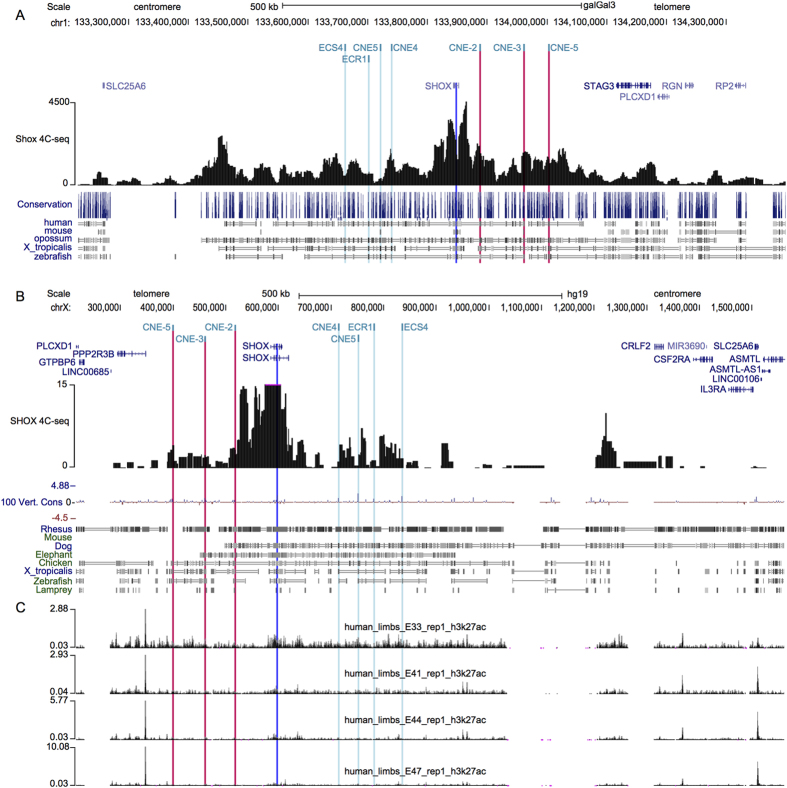
4C-seq chromatin interaction map of the *cis*-regulatory region surrounding *SHOX*. (**A**) 4C-seq data of the chicken *Shox* locus (chr1:133,215,000-134,400,000; UCSC, galGal3) showing that the *Shox* promoter (blue bar, viewpoint) interacts with 800 kb of adjacent genomic regions that likely contain *Shox cis*-regulatory regions, a region syntenic with about 1 Mb of human genomic DNA (shown in panel (**B**)). Red bar marks the position of the upstream CNEs. The 4C-seq data indicate that the *Shox* regulatory landscape extends both upstream and downstream of the currently known, functionally validated enhancers. (**B**) 4C-seq data of the human *SHOX* locus (chrX:215,733–1,561,244; UCSC, hg19) showing that the *SHOX* promoter (blue bar, viewpoint) interacts with the upstream enhancers (red bars). The RefSeq Genes are shown a the top and the conservation track is included at the bottom of panel (**B**). (**C**) Distribution of enhancer-associated H3K27ac histone marks at different human embryonic limb bud stages, produced by Cotney *et al*. (2013).

Several genomic alterations associated with *SHOX*-deficient phenotypes have been found within regulatory domains both upstream and downstream of the *SHOX* coding region^15^,^16^,^20^,^21^,^22^. Subsequent functional studies identified seven CNEs with enhancer properties in these regions and 3C analysis showed the interaction of downstream elements with the *Shox* promoter^5^,^6^,^7^,^8^, suggesting a complex regulation of *SHOX*. To further map the *cis*-regulatory landscape of *Shox*, we used 4C-seq, a chromosome conformation capture technique that has been used to identify *cis*-regulatory elements associated with *Hox* genes^29^,^30^,^31^. Using the *Shox* promoter as an anchor point, 4C-seq in chicken limb buds revealed widespread contacts between *Shox* and multiple upstream and downstream regions. The region extended almost 800 kb in the chicken genome, a region syntenic with approximately 1 Mb of human genomic DNA (Fig. 5A). All three known upstream enhancers (CNE-5, CNE-3 and CNE-2) contacted with the *Shox* promoter. Similar results were generated using 4C-seq in human U2OS cells (Fig. 5B). Interestingly, integration of the previously generated H3K27ac ChIP-seq marks in the *SHOX* locus with the 4C-seq peaks revealed numerous other active regions, upstream and downstream of the *SHOX* transcription unit (Fig. 5C). These findings suggest a complex regulation of *SHOX* expression by multiple *cis*-regulatory elements located upstream and downstream of the coding region within a large genomic region extending beyond 1 Mb (approximate boundaries chrX:284,600-1,355,600, hg19).

## Discussion

CNVs of *cis*-regulatory elements of strict spatiotemporally regulated transcription factor genes can often underlie human developmental disease, as is the case for *SHOX*, which is regulated by at least seven enhancers located downstream and upstream of *SHOX*^5^,^6^,^7^,^8^. Indeed, enhancer activity of the upstream CNEs (CNE-5, CNE-3, CNE-2) has been previously demonstrated by *in ovo* enhancer assays in chicken limb buds, however, specific functional data in relevant human cells are lacking. We therefore performed luciferase assays in human U2OS cells, showing increased luciferase activity for all three upstream CNEs in a localization-independent manner, confirming their enhancer activity in human cells. In addition, the upstream CNEs overlap with previously identified active enhancer-associated histone H3K27ac marks in human limbs of different developmental stages^10^. *Cis*-regulatory elements can only fulfil their function if the chromatin is in an open euchromatic conformation and if they can communicate with the promoter of a target gene^4^. The most favoured model to explain such communication is the looping model where transcription factors bound at the enhancer make direct contact with the promoter and/or with factors bound at the promoter, while the intervening DNA loops out^32^. This model forms the basis for the 3C/4C methods, which allow the identification of interactions between genomic fragments^33^. To test the interactions of the upstream enhancers, we performed 4C-seq in limb buds of chicken embryos and in human U2OS cells. Both experiments demonstrated interactions for all three upstream CNEs and the *Shox* promoter, emphasizing their *cis*-regulatory role in both chicken and human cells.

*SHOX* deficiency can result from a variety of molecular defects including intragenic *SHOX* mutations, complete and partial CNVs of *SHOX,* and CNVs of its enhancers located in the downstream PAR1 region^1^,^12^,^13^,^14^,^15^,^16^,^17^,^18^,^19^,^20^,^21^. The first CNVs of the upstream enhancer, a deletion and a duplication, were only recently identified in ISS patients^21^,^22^. As the upstream region has only been included relatively recently in the most frequently used diagnostic tool for CNV analysis of *SHOX* and the PAR1 region (i.e. multiplex ligation-dependent probe amplification, MLPA), CNVs of the upstream enhancers may have been missed and therefore their contribution to the pathogenesis of *SHOX*-deficient phenotypes might have been underestimated. Here, CNV analysis of the upstream CNEs in 501 cases with unexplained ISS revealed two upstream enhancer deletions and one duplication. Thus, the frequency of upstream CNVs in our ISS cohort is only ~0.6% while an even lower frequency was reported by Benito-Sanz *et al*.^22^. These low numbers are in contrast with the high frequencies observed for downstream CNVs. This might have several explanations: (i) Differences in the genomic content between the upstream and downstream region. Indeed, Durand *et al*. showed that the overall content of interspersed repeats of the downstream region is considerably higher than that of the upstream region thus increasing the probability of deletions and duplications^7^. (ii) Lack of screening studies of the upstream CNEs in large ISS cohorts as only this and the one reported by Benito-Sanz *et al*.^22^ have been undertaken to date. (iii) Smaller phenotypic effects of CNVs of upstream CNEs compared to those of downstream CNVs, which could be due to different spatiotemporal regulation, may lead to milder phenotypes that are not necessarily ascertained at genetic and endocrinology clinics^22^. In addition this could explain incomplete penetrance, as assumed for the upstream CNV detected in family A and for the previously reported upstream duplication^21^. Interestingly, incomplete penetrance was also observed in several families with the recurrent ~47.5 kb downstream deletion^8^, suggesting that CNVs of the upstream or downstream CNEs can act as rare functional variants that require (an) additional risk factor(s) to express their phenotypes. This is also supported by the relatively high number of CNVs encompassing one or more upstream CNEs found in DGV and Decipher.

Our data underscore that the clinical significance of upstream CNVs, especially duplications, is often ambiguous, hampering genetic counselling. The orientation and location of the duplicated segment, may cause overexpression of its target gene or interfere with the chromatin conformation, thereby impairing *cis*-regulation during development and causing disease^34^.

In ~60–80% of LWD and ~2–5% of ISS cases a *SHOX*-related genetic defect can be identified, leaving a large proportion of LWD and ISS cases molecularly unexplained. Besides the involvement of variants in other loci, genetic defects in novel, as yet unidentified *cis*-regulatory elements of *SHOX* may explain a proportion of these unexplained LWD and ISS cases. In order to delineate the *cis*-regulatory domain more precisely, we performed 4C-seq in different cells. Interestingly, the chromatin interaction maps delineate the *cis*-regulatory landscape of *SHOX* to a region that extends beyond 1 Mb in humans (approximate boundaries: chrX:284,600–1,355,600, hg19), which is larger than previously anticipated based on the genomic positions of the previously functionally studied CNEs (approximate boundaries: chrX:398,357–835,567, hg19). These data are consistent with the multiple enhancer-associated H3K27ac marks in human limbs distributed along the whole region and active at different developmental stages^10^. The boundaries proposed here appear to be larger than the topological domain (chrX:350,001–1,035,000) determined by Hi-C in GM12878 lymphoblastoid cells, (Supplementary Fig. S4)^35^. These differences can be explained by the presence of several assembly gaps in the *SHOX* region for which consequently no Hi-C data can be generated. Taking this into account, the regulatory domains mapped both by Hi-C and 4C-seq do have similar boundaries. This also illustrates that the spatial organisation of the genome in topological domains is stable across different cell types and is highly conserved throughout evolution^36^.

Integration of the 4C-seq interaction map generated here and H3K27ac histone marks will be instrumental for the identification of novel *cis*-regulatory elements. In this context, it is interesting to mention two recently reported deletions ~300 kb downstream of *SHOX* in LWD, not including any of the four known downstream enhancers^37^,^38^. These deletions harbour a high 4C-seq interaction peak and several H3K27ac marks possibly pointing to a novel *SHOX* enhancer (Supplementary Fig. S5). Further *in vitro* and *in vivo* assays are required however to substantiate this.

In conclusion, *in vitro* luciferase assays of upstream CNEs in human U2OS cells and active histone marks in human developing limbs support an enhancer role for the upstream CNEs in human cells. Chromosome conformation capture profiling (4C-seq) in embryonic chicken limb buds and human U2OS cells show *cis*-regulatory interactions of the upstream CNEs with the *SHOX* promoter. In addition, our study revealed a low frequency of upstream CNVs in a sizeable ISS cohort. Finally, the 4C-seq interaction and H3K27ac ChIP-seq profiles revealed that the *cis*-regulatory domain of *SHOX* extends beyond 1 Mb surrounding *SHOX*. Thus, novel *cis*-regulatory elements may be located upstream and downstream of *SHOX*, of which variations might contribute to *SHOX*-deficient phenotypes.

## Materials and Methods

### Luciferase assays

The enhancer reporter constructs contained the upstream enhancers, CNE-5, CNE-3 and CNE-2 upstream of the human *SHOX* promoter (−432 to +5 bp, NM_000451). The sequences including the upstream enhancers were amplified using the following primers: 5′- CAAACACGGAACAGCACACT -3′ and 5′- CCTGGGACAGACACGACC -3′ for CNE-5, 5′- CGAGGTGGATCAAAGTG -3′ and 5′- TGCTCTGCCATATCCTCAATC -3′ for CNE-3, and 5′- ACATGACAGCCGGGCCTCTG -3′ and 5′- GCGAGCCATAAAACAAGCTG -3′ for CNE-2, and cloned into the pGL3SHOXprom vector, upstream of the promoter (8), thus mimicking its chromosomal localization. The enhancers were also cloned downstream of the luciferase gene to test out localization dependency. The enzymes utilized were KpnI and NheI for cloning the enhancers upstream of the *SHOX* promoter and BamHI and SalI for cloning downstream of the luciferase gene. Luciferase assays were undertaken in human U2OS osteosarcoma cells, as previously reported^8^. The downstream *SHOX* enhancers, CNE4, CNE5, ECR1 and ECS4/CNE9^6^,^8^ were also cloned and employed as positive controls while ECS5^5^ and the empty pGL3SHOXprom vector (i.e. no enhancer) were used as negative controls. The data shown is the mean and standard deviations of three replicates of each transfection and two biological replicates, thus a total of six points for each enhancer.

### Patient cohort

A total of 501 probands with ISS were referred to the Center for Medical Genetics in the Ghent University Hospital for genetic testing of the *SHOX* region. An informed consent was obtained from all subjects. Revisiting of clinical records was performed in cases in whom an upstream CNV was found. Whenever possible, clinical data included: sex, age, birth details, height standard deviation score (SDS) according to national standards, physical examination of extremities, X-rays of the lower arm and family histories including parental heights SDS (Table 1). The study was conducted in accordance with the tenets of Helsinki and was approved by the Ghent University Hospital Ethics Committee (approval 2004/094).

### Pre-screening of *SHOX* and the neighbouring PAR1 region

Prior to this study, all cases were genotyped using our previously described PAR1 qPCR-based copy number analysis test^18^. As coding mutations of *SHOX* were excluded in only 56 of these cases by Sanger sequencing, the remainder of patients underwent in-house developed targeted next-generation sequencing (NGS) of the coding exons and intron-exon boundaries of *SHOX* (MiSeq, Illumina, San Diego, CA)^23^. Primers and PCR conditions used for the amplification of the coding regions of *SHOX* are available upon request.

### Upstream qPCR-based copy number profiling

CNV analysis was performed as previously described with minor modifications (Supplementary Data S6)^18^. Three amplicons, one for each upstream CNE (CNE-5, CNE-3 and CNE-2), were designed and subjected to stringent *in silico* and *in vitro* validations to guarantee their specificity and efficiency^39^. Next, qPCR reactions were carried out on the LightCycler 480 Instrument II (Roche Applied Science, Penzberg, Germany) and data-analysis was subsequently performed with the commercially available qBasePlus software (Biogazelle NV, Zwijnaarde, Belgium)^40^. Two reference genes, *ZNF80* and *GPR15*, were used for normalization of the relative quantities and three positives controls with known copy number were used as a reference to calculate the copy numbers.

### Array-based comparative genomic hybridization (arrayCGH)

Genome-wide copy number profiling was performed on 180 K oligonucleotide arrays (Agilent Technologies, Santa Clara, CA). In addition, a custom high-resolution 8 × 60 K Agilent microarray was designed using the online design tool eArray, targeting a region of 693 kb around *SHOX* (chrX:247,599–940,876; UCSC, Human Genome Browser, hg19). Hybridizations were performed according to manufacturer’s instructions with minor modifications. The results were subsequently visualized in arrayCGHbase^41^.

### Chromosome conformation capture assays (4C-seq)

The experimental chicken procedures have been performed following the protocols approved by the Ethical Committee for Animal Research from Consejo Superior de Investigaciones (CSIC) according to the European Union regulations. The 4C-seq assay was performed as previously reported^33^,^42^,^43^,^44^. HH28 chicken embryos were dissected and limbs were processed to obtain approximately 10 million isolated cells. Human U2OS cells were processed to obtain 4.5 million cells. Human and chicken samples were equally processed. Cells were lysed (lysis buffer: 10 mM Tris-HCl pH 8, 10 mM NaCl, 0.3% IGEPAL CA-630 (Sigma-Aldrich, St. Louis, MO), 1X protease inhibitor cocktail (Roche Applied Science), the DNA digested with DpnII endonuclease (New England Biolabs, Ipswich, MA) and ligated with T4 DNA ligase (Promega). Subsequently, Csp6I endonuclease (Thermo Fisher Scientific, Waltham, MA) was used in a second round of digestion, and the DNA was ligated again. Specific primers were designed at the *Shox* promoter with Primer3 v. 0.4.0^45^ (Supplementary Data S7). Illumina adaptors were included in the primers sequence and 16 PCRs were performed with Expand Long Template PCR System (Roche Applied Science) and pooled together. This library was purified with a High Pure PCR Product Purification Kit (Roche Applied Science); its concentration measured using the Quanti-iTTM PicoGreen dsDNA Assay Kit (Life technologies) and sent for deep sequencing. The 4C-seq data were analysed as previously described^43^. Briefly, chicken and human raw sequencing data were de-multiplexed and aligned using the Chicken May 2006 assembly (galGal3) and Human February 2009 (hg19) respectively, as the reference genome. Reads located in fragments flanked by two restriction sites of the same enzyme, or in fragments smaller than 40 bp were filtered out. Mapped reads were then converted to reads-per-first-enzyme-fragment-end units, and smoothed using a mean running window algorithm. Data was submitted to GEO under accession GSE65959.

## Additional Information

**How to cite this article**: Verdin, H. *et al.* Profiling of conserved non-coding elements upstream of *SHOX* and functional characterisation of the *SHOX cis*-regulatory landscape. *Sci. Rep.* **5**, 17667; doi: 10.1038/srep17667 (2015).

## Supplementary Material

Supplementary Files

## Figures and Tables

**Table 1:** Anthropometric and phenotypic characteristics of the probands with an upstream CNV and their parents.

Patient	CNV	Age	Ethnicity	Gender	Anthropometric measurements (SDS)	Birth details	Parental anthropometric measurements (SDS)	Other clinical characteristics
A	~91 kb (arr[hg19] Xp22.33(426,377–517,515)x1 pat)	9 yrs	Caucasian	F	115.8 cm (−2.3 SDS)	Born at full term with a birth weight of 3.08 kg and birth length of 50 cm	Father: 182 cm (0.55 SDS) Mother: 163 cm (−0.14 SDS)	X-rays of the wrists and lower arms were normal as were thyroid test and IGF-I levels. Arm span:height ratio was 0.95.
B	~139 kb (arr[hg19] Xp22.33(345,956–485,020)x1)	13 yrs	Roma	M	130.5 cm (−2.5 SDS)	ND	Father: 170 cm (1.03 SDS) Mother: 152 cm (–1.96 SDS)	ND
C	~256 kb (arr[hg19] Xp22.33(283,986–539,708)x3 pat)	17 yrs	Caucasian	F	153 cm (−2.3 SDS)	Born at full term with a birth weight of 3.1 kg and birth length of 49 cm	Father: 163 cm (−2.6 SDS) Mother: 156 cm (−1.8 SDS)	X-rays of hand and wrist were normal. Shorter proximal extremities and clinodactyly of the fifth fingers.

Abbreviations used (in alphabetical order): CNV: copy number variation; F: female; M: male; ND: not documented; SDS: height standard deviation score; yrs: years.
