# Supplementary material for: Profiling of conserved non-coding elements upstream of SHOX and functional characterisation of the SHOX cis-regulatory landscape
Source: Sci Rep. 2015 Dec 3;5:17667. doi: 10.1038/srep17667 (PMC4668379; doi:10.1038/srep17667)
Supplement: Supplementary Files [file srep17667-s1.pdf]

Profiling of conserved non-coding elements upstream of *SHOX* and functional characterisation of the *SHOX cis*-regulatory landscape

Hannah Verdin[1], Ana Fernández-Miñán[2], Sara Benito-Sanz[3,4], Sandra Janssens[1], Bert Callewaert[1], Kathleen De Waele[5], Jean De Schepper[5], Inge François[6], Björn Menten[1], Karen E. Heath[3,4], José Luis Gómez-Skarmeta[2¶], Elfride De Baere[1*¶]

Supporting Information

Supplementary Figure S1. ArrayCGH profiles of the upstream deletions (A and B) and duplication (C).

Supplementary Figure S2. Copy number profiling of the upstream CNEs in the parents.

Supplementary Table S3. CNVs overlapping the upstream CNEs reported in DGV, Decipher and our local database.

Supplementary Figure S4. Topological domain of the *SHOX* locus

Supplementary Figure S5. Overview of novel downstream deletions not overlapping with known *cis*-regulatory elements.

Supplementary Data S6. qPCR primers and conditions for copy number screening of *SHOX*.

Supplementary Data S7. Primers used for 4C-seq analyses in chicken and human.

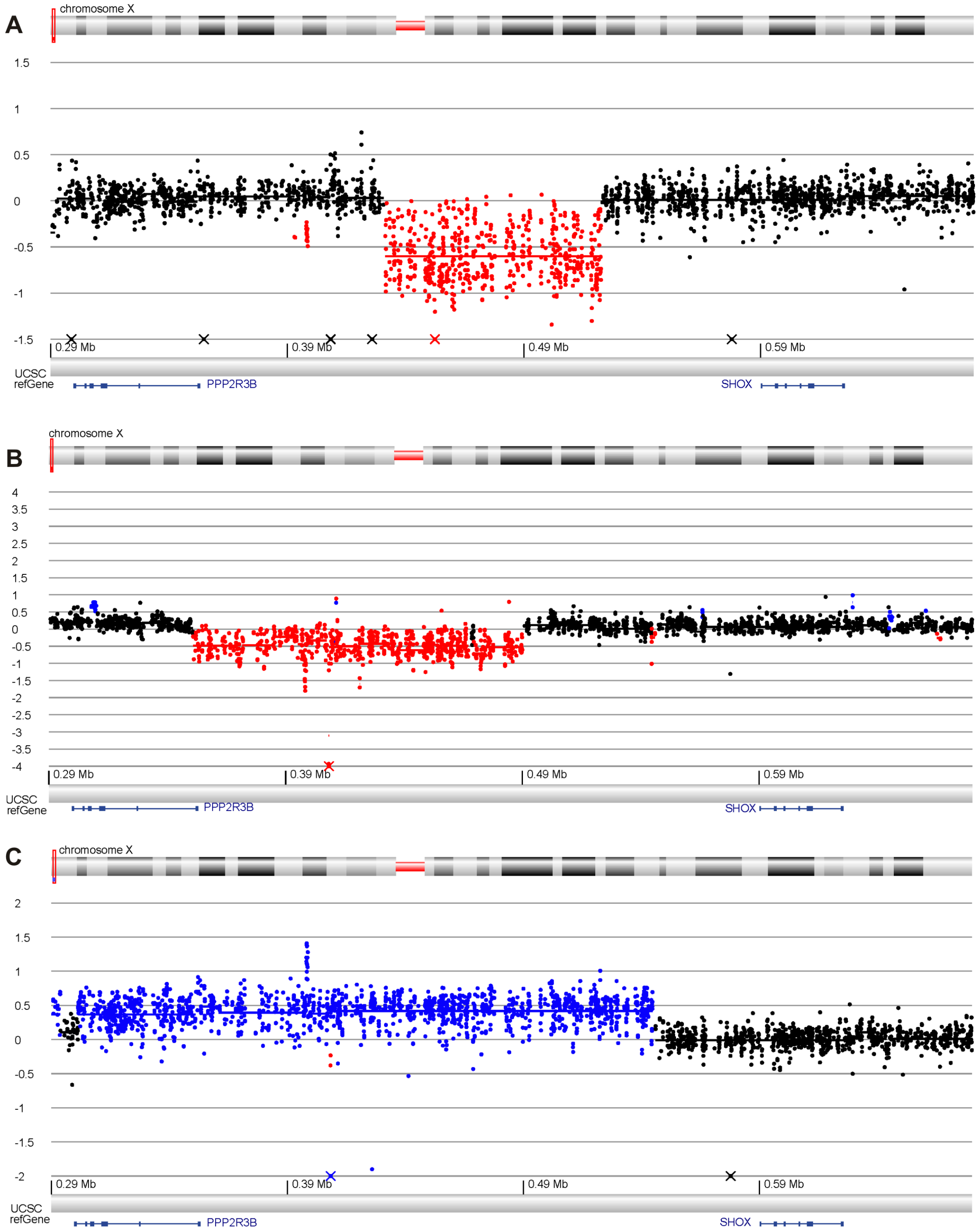

Supplementary Figure S1. ArrayCGH profiles of the upstream deletions (A and B) and duplication (C). At the top, for reference, chromosome X is represented with a red rectangle indicating the location of the displayed array profile. At the bottom, the genomic position is shown in more detail. The red (loss), blue (gain) and black (no change) dots represent $\log_2$-ratios of individual oligonucleotides. The crosses in the bottom line represent non-hybridized probes.

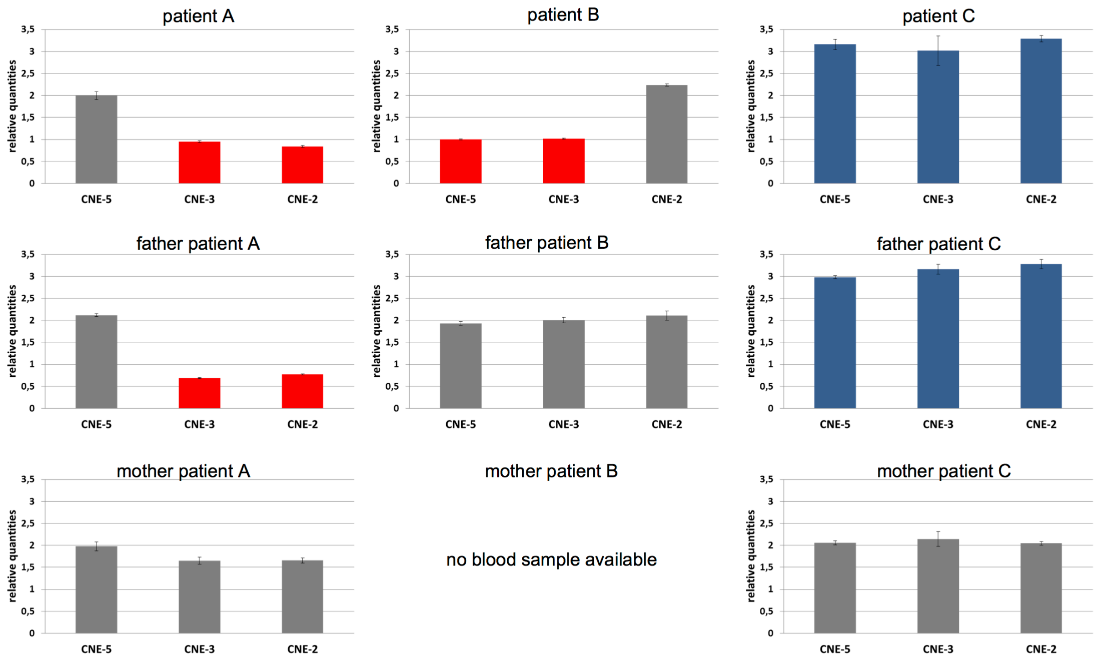

Supplementary Figure S2. Copy number profiling of the upstream CNEs in the parents. Each panel shows the results of the copy number profiling using qPCR. The qPCR-derived copy number results are presented as relative quantities in a bar chart. Normal copy numbers are represented in grey, deletions are shown in red and duplications in blue. Error bars are added to allow interpretation of the assay's precision. The analysis could not be performed for the mother of patient B as no blood sample was available.

Supplementary Table S3. CNVs overlapping the upstream CNEs reported in DGV, Decipher and our local database.

CNVs in DGV overlapping chrX:398,357-517,229

DGV ID	Variant	Genomic position (hg19)	Interval (Mb)	Reference
esv2972679	Gain	chrX:368000-436000	0.068	Pang et al., G3, 2013
esv2973790	Gain	chrX:454000-472000	0.018	Pang et al., G3, 2013
nsv508745	Gain	chrX:374076-534155	0.16	Teague et al., Proc Natl Acad Sci U S A, 2010
esv2758854	Gain	chrX:343529-562251	0.22	Redon et al., Nature, 2006

CNVs in our local arrayCGH database overlapping chrX:398,357-517,229

Patient	Variant	Genomic position (hg19)	Interval (Mb)	Phenotypes
14-year old boy	Loss	chrX:431793-463800	0.032	Short stature (-3,9 SDS), intellectual disability, delay in motor development and joint hyperlaxity
1-year old girl	Gain	chrX:219609-537,091	0.317	Infant respiratory distress syndrome with pulmonary hypertension and persisting ductus arteriosus, short stature (-3,5 SDS), neuromotor delay
6-year old girl	Gain	chrX:155490-537,240	0.38	Neuromuscular problems with absent reflexes of the lower limbs, amyopathic face, joint laxity and hyperlordosis

CNVs in Decipher overlapping chrX:398,357-517,229

Decipher ID	Variant	Genomic position (hg19)	Interval (Mb)	Classification in Decipher	other CNVs	Phenotypes
289796	Gain	chrX:166314-416347	0.25	Likely benign	Gain, X:8268774-8391934	Autism
288467	Gain	chrX:168992-414716	0.25	Likely benign	Loss, 22:50937280-51154705	Intellectual disability, delayed speech and language development
288571	Gain	chrX:168992-506573	0.34	Likely benign	5 others	Intellectual disability
288691	Gain	chrX:179394-437248	0.26	Likely benign	Loss, 22:50937280-51154705	-
284256	Gain	chrX:284015-506124	0.22	Uncertain	Gain, 1:235371503-235597612	Intellectual disability
289744	Gain	chrX:286545-406537	0.12	Likely benign	Gain, Y:234863-356537	Intellectual disability, macrocephaly
288905	Gain	chrX:286545-460331	0.17	Possibly pathogenic	Loss, 6:162039650-162293992	Global developmental delay, obesity, tachycardia, preauricular pit, strabismus, self-mutilation, absent speech
287930	Gain	chrX:286545-520629	0.23	Likely benign	Gain, Y:236545-470629	Seizures
287896	Gain	chrX:287576-402002	0.11	Likely benign	Gain, Y:242096-352002	Generalized seizures, familial predisposition
288758	Gain	chrX:296482-500404	0.20	Possibly pathogenic	Gain, Y:246482-450522	Autism
287963	Gain	chrX:296482-520629	0.22	Likely benign	Gain, 22:17393188-17454928	Intellectual disability, mild, abnormal emotion/affect behavior
289812	Gain	chrX:316361-584438	0.27	Possibly pathogenic	-	Generalized seizures
265981	Gain	chrX:324303-492278	0.17	Unknown	-	Secundum atrial septum defect, coarctatio of aorta, exotropia, moderate global developmental delay, upslanted palpebral fissure, anteverted nares, highly arched eyebrow, low-set ears
290063	Gain	chrX:329874-432599	0.10	Likely benign	-	Microcephaly
290233	Gain	chrX:329946-520629	0.19	Likely benign	Gain, Y:266361-470629	Intellectual disability, behavioral/psychiatric abnormality
289860	Gain	chrX:387593-582903	0.20	Possibly pathogenic	Gain, Y:337593-535239	Spastic diplegia
286155	Gain	chrX:455566-544731	0.09	Definitely pathogenic	Gain, 16:29563985-30107008	Global developmental delay, overgrowth, macrocephaly
288006	Gain	chrX:486881-537040	0.05	Likely benign	3 others	Short stature, cryptorchidism, defect in the atrial septum
290125	Gain	chrX:486881-537040	0.05	Likely benign	2 others	Intellectual disability
286107	Loss	chrX:455566-544731	0.09	Definitely pathogenic	2 others	Global developmental delay, seizures, inverted nipples, generalized hypotonia

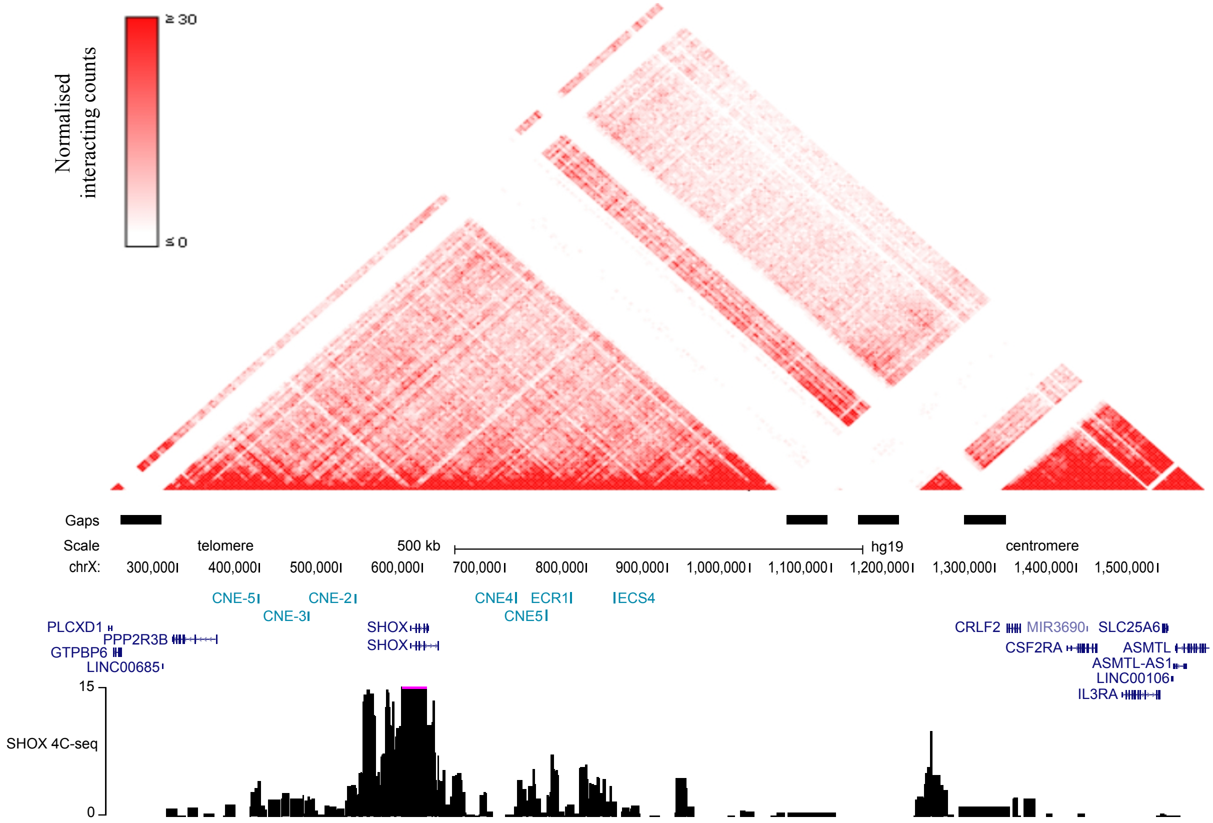

Supplementary Figure S4. Topological domain of the *SHOX* region. Overview of the *SHOX* region (hg19: chrX:215,733-1,561,244; UCSC, Human Genome Browser, hg19) showing a two-dimensional heat map representing normalised Hi-C interaction frequencies in the GM12878 cell line at a resolution of 5 kb generated by Rao et al. (2014). UCSC tracks display the gaps in the assembly (hg19), the known validated enhancers (CNEs), the RefSeq Genes and the 4C-seq interaction peaks.

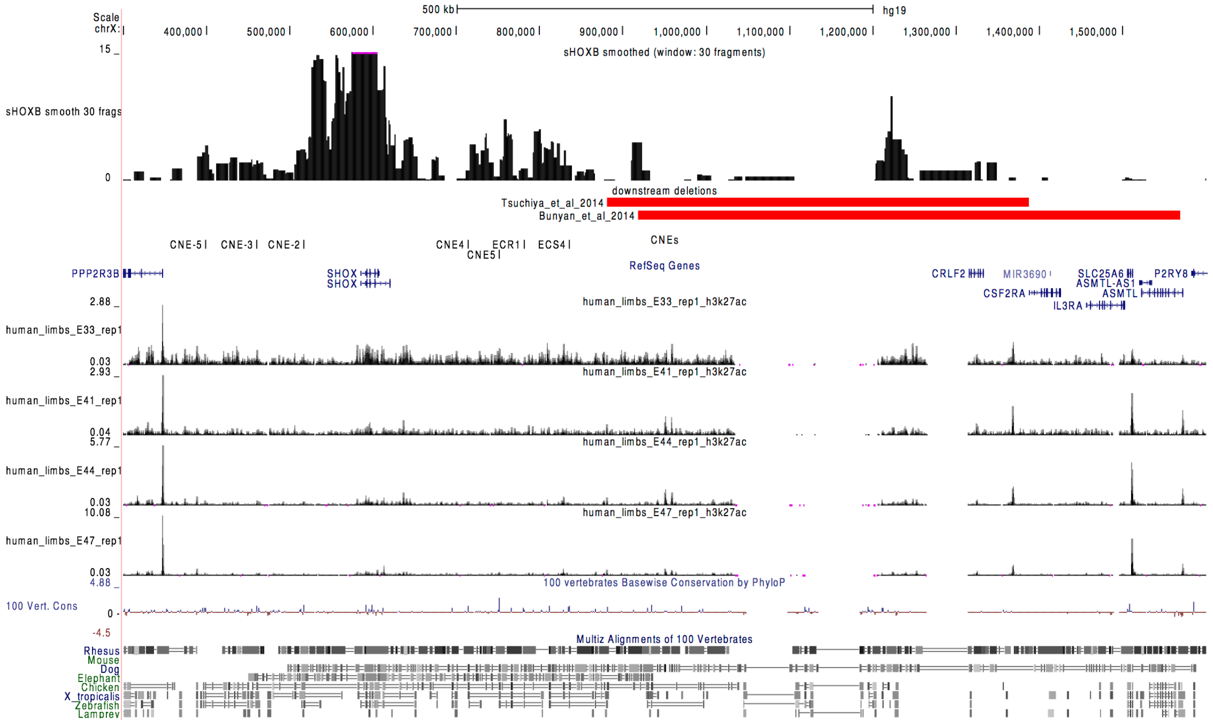

Supplementary Figure S5. Overview of novel downstream deletions not overlapping with known *cis*-regulatory elements. Overview of the *SHOX* region (hg19: chrX:300,000-1,600,000; UCSC, Human Genome Browser, hg19) showing the 4C-seq interaction data at the top, the novel downstream deletions (red bars), the known validated enhancers (CNEs), the RefSeq Genes, the enhancer-associated H3K27ac histone marks at different human embryonic limb bud stages, produced by Cotney et al. (2013) and the conservation track.

Supplementary File S6. qPCR primers and conditions for copy number screening of *SHOX*.

qPCR primers:

Amplicon	Forward primer (5’ – 3’)	Reverse primer (5’ – 3’)	Amplicon	Genomic position (hg19)
CNE-5	tcccggccttccttcttct	tgccctgcctcttccatct	164	chrX:398379-398542
CNE-3	ttctgtgcccggagatcaaagt	cgtgcagagctcaaggtgtcata	156	chrX:460407-460562
CNE-2	cttcggagaatgcagcgaccatat	gtttctctcttccctccttctctccttta	120	chrX:516707-516826

qPCR conditions:

- 10 ng DNA
- 2x SsoAdvanced™ SYBR® Green Supermix (Bio-Rad, Hercules, CA)
- 5 µM of each primer (IDT, Coralville, IA)

qPCR run protocol

qPCR step	Temperature	Time
Pre-incubation	98°C	2 min
40 amplification cylces	98°C	5 sec
	60°C	30 sec
Cooling	40°C	30 sec

Supplementary File S7. Primers used for 4C-seq analyses in chicken and human.

1. **4C-seq in embryonic chicken limbs**

Viewpoint

chr1:133,851,004-133,851,680 (galGal3)

Primers used for 4C-seq analysis

Reading primer: shox_gal_4C_dpnII_RS_rev	GCCGCTTCCTTCTGTTTGATC
Non-reading primer: shox_gal_4C_csp6I_NRS_fwd	GAAGTTGCGGCTCTTTCC

With Illumina adaptors

shox_gal_4C_dpnII_RL:
AATGATACGGCGACCACCGAACACTCTTTCCCTACACGACGCTCTTCCGATCTGCCG
CTTCCTTCTGTTTGATC

shox_gal_4C_csp6I_NRL: CAAGCAGAAGACGGCATACGAGAAGTTGCGGCTCTTTCC

2. **4C-seq in human U2OS cells**

Viewpoint

chrX:585,213-588,229 (hg19)

Primers used for 4C-seq analysis

Reading primer:
SHOX_hg_4C_dpnII_RS_rev GTTGGAGTTTTACAGAAAGATGAGATC

Non-reading primer:
SHOX_hg_4C_csp6I_NRS_fwd AGGGTCTGCACCTCTCTTGG

With Illumina adaptors

SHOX_hg_4C_dpnII_RL:
AATGATACGGCGACCACCGAACACTCTTTCCCTACACGACGCTCTTCCGATCTGTTG
GAGTTTTACAGAAAGATGAGATC

SHOX_hg_4C_csp6I_NRL:
CAAGCAGAAGACGGCATACGAAGGGTCTGCACCTCTCTTGG
